# Supplementary material for: Quality of sleep and associated factors among people living with HIV/AIDS attending ART clinic at Hawassa University comprehensive specialized Hospital, Hawassa, SNNPR, Ethiopia
Source: PLoS One. 2020 Jun 4;15(6):e0233849. doi: 10.1371/journal.pone.0233849 (PMC7272010; doi:10.1371/journal.pone.0233849)
Supplement: S1 Table — (DOCX) [file pone.0233849.s001.docx]

S1 Table: Sleep quality and its components score of PLWHA attending ART clinic at HUCSH, SNNPR, Ethiopia, 2019 (n=389) (DOCX)

| PSQI components | Mean (SD) | |
| --- | --- | --- |
|  | Poor | Good |
| C-1 Self-rated sleep quality (0-3 score) | 1.17 (+0.51) | 0.65(+0.52) |
| C-2 Time it takes to fall asleep (min) | 58.57 (+49.02) | 23.9(+12.3) |
| C-3 Total hours of sleep per night (hr.) | 6.7 (+5.6) | 8.2(+6.2) |
| C-4 Sleep efficiency (%) | 74.3 (+13.74) | 96.4(+86.9) |
| C-5 Sleep disturbances (0-27 score) | 9.85 (+4.57) | 6.05(+3.97) |
| C-6 Need for sleep medication (0-3 score) | 0.33 (+0.7) | 0.08(+0.29) |
| C-7 Day time dysfunction (0-3 score) | 1.0 (+0.97) | 0.3(+0.6) |
| Total PSQI score | 8.43 (+2.74) | 3.21 (+1.47) |

**N.B:** Sleep efficiency= Total hours of actual sleep per night/Total hours in bed per night x100
